# Foreign

**Published:** 1841-07-31

**Authors:** 


### FOREIGN.

*Colica Pictonam treated with Warm Water.* By JOHN WILSON, Physician to the Middlesex Hospital.—In this paper the author, after referring to the complicated treatment of the disease pursued at the Hospital of La Charité, proposes to illustrate two of the remedies there in combination, namely, enemas and hot baths. In six cases of colica pictonum, some of them of much severity, and complicated with paralysis, Dr. Wilson has employed enemata, administered in the hot bath, in one case by the patient himself, and consisting of the water of the bath.

The effect of this combination has been very successful, both in regard to the symptoms of pain and constipation, and to the paralysis, where that existed. In the course of his statement, the author notices the presence of looseness of the gums, blueness at their edges, and a fœtor like that of mercury, when none had been taken; and informs us, that he has noticed this latter circumstance in other cases of colica pictonum.

He next details a case of constipation, not attributable to lead, in which the above remedies had proved equally successful.

In some of the above cases the treatment is used alone, in others it is followed up by doses of the soap and opium pill; of castor-oil with tincture of opium; or with a solution of sulphate of magnesia and carbonate of magnesia in mint-water.—*Lancet.*

This is a novel, but we do not doubt a successful mode of treatment, and would very probably prove effectual in the management of other cases of colic.

*On Asphyxia from Carbonic Acid.* By M. Devergie.

In the 11th number of this journal, our readers will find the report of a singular case of murder by carbonic acid, reported by M. Devergie. Some of the medico-legal questions which were raised in that case were of so novel a character, that the author has been induced to re-examine the whole subject. We shall here give an abstract of the results of his observations and experiments.

1. *On the circumstances under which asphyxia by carbonic acid may take place.* It is commonly supposed that asphyxia by carbonic acid is not likely to occur in those cases, where the source of combustion does not exist in the apartment itself in which an individual may be found apparently dead. This is an error, for it is well known that when two chimneys or the flues of stoves communicate, if a fire be lighted in one room, the vapour may be drawn through the communicating chimney by a downward current into a distant room. The distribution of the vapors of burning fuel will therefore depend greatly upon the state of expansion or contraction in the air of such communicating apartments. This source of asphyxia has been hitherto overlooked. M. D'Arcet has published several cases which show that serious accidents may thus occur without the cause being even suspected by the medical attendant.

2. *What takes place in an apartment in which a charcoal fire has been lighted?* When the charcoal is kindled, and so long as it burns, it absorbs oxygen from the air, producing thereby carbonic acid and carburetted hydrogen: the latter, however, is converted as soon as it is formed into carbonic acid and water. Since the carbonic acid thus produced occupies the same bulk as the oxygen which was required for its production, it follows that the quantity of air in the apartment can undergo no augmentation, if we except the addition to it of a very small portion of carburetted hydrogen that may escape combustion. But, although there is no actual increase in quantity, there is a vast difference in bulk, which is due to the great expansion caused in gases by a rise of temperature. The heat of combustion is such, as probably to give a threefold volume to the air of an apartment, and to cause the upper and lower strata rapidly to change places. This increased bulk must continue during the whole period of combustion; and the dilated air will endeavour to find an outlet through every fissure or opening connected with the apartment. If there be any impediment to this escape, it will exert a pressure in proportion to its state of expansion on all surrounding bodies; a pressure which would materially impede respiration, and accelerate the access of asphyxia.

There is another fact of medico-legal interest. While the combustion lasts, there are two currents of air circulating through the room; the one ascending of heated air, and therefore rich in carbonic acid, the other descending of cold air. It is by means of the ascending current that the carbonic acid produced in combustion is equally diffused through all parts of the chamber. To establish this point, several experiments were made. Among others, two vessels for collecting gases were opened in the apartment, so soon as the combustion had terminated; the one being placed at the top, and the other on the floor. The quantity of carbonic acid collected was perceptibly the same in the two cases, the proportion being about one-fiftieth part. Hence it follows that during the combustion of charcoal, the carbonic acid is equally diffused throughout the space in which it is produced.

The air of such an apartment is unfit for respiration: 1st, because it is deficient in the quantity of oxygen necessary for the maintenance of that function; and, 2d, because it is contaminated by deleterious gases.

So soon as the combustion is finished, there is a tendency to the production of an equilibrium in temperature; the expanded air slowly contracts, and occupies less bulk. If any air had previously escaped from the apartment through openings, a fresh portion now enters to supply its place, so that when the equilibrium is perfect, the air is less vitiated than it was during combustion. If the apartment were so close that no air could escape, then the pressure of the expanded mass diminishes with the decrease of temperature, and resumes its original condition; but, in this case, it is just as much contaminated as it was while the charcoal was burning.

3. *Do the gases formed separate according to their specific gravities after the air has cooled?* This is a question of material importance in relation to asphyxia, and to illustrate its application, the following supposed case may be taken: Two persons are found asphyxiated in the same chamber: the one may be stretched on the bed, the other lying on the floor. Which of these two has been more powerfully affected by the asphyxiating cause, (the charcoal vapour?) The solution of this question must rest partly on reasoning and partly on experience. After the complete combustion of charcoal, the respirable air becomes specifically lighter: a large quantity of oxygen has been removed from it in the production of carbonic acid; nitrogen now predominates, and this gas is lighter than air. Hence we may affirm that irrespective of the carbonic acid produced, the specific gravity of the air of the chamber has diminished. The specific gravity of carbonic acid is one-half greater than that of air, (1·5:1;) but in consequence of the change which the air in the chamber has undergone, the difference becomes still greater. We might therefore infer that when once the air has cooled, or even during the act of cooling, the carbonic acid would tend to fall to the lowest part of the chamber. To this inference may be opposed the experiments of Dalton on the diffusion of gases, which establish that these bodies, notwithstanding the difference in their specific gravities, have a tendency to mix uniformly on contact. M. Devergie was, however, induced to think that under the above-mentioned circumstances, carbonic acid would collect on the floor of an apartment, in a stratum of variable density; and to remove any doubt on the point, the following experiment was performed:

Two vessels proper for collecting gas were placed, the one at the highest and the other at the lowest part of a moderately large chamber, in which a quantity of charcoal was burnt. The next day, when the apartment had become thoroughly cooled, the bottles of gas were removed; a quantity of solution of potash was put into each, and they were immediately closed. Two hours afterwards, the solution was placed in a proper vessel connected with a graduated jar, and sulphuric acid added until the potash was saturated. The liquid in the bottle from the floor of the chamber yielded 150 parts of carbonic acid; while the other vessel, which was at the summit of the apartment, gave only 32 parts. A cat placed on the floor uttered painful cries for an hour and a half, and the next morning was found dead.

We must therefore consider it as proved by this experiment, that after combustion has ceased, and while the apartment is cooling, the carbonic acid *separates from the air by its greater specific gravity*, and forms on the floor a stratum, of which the thickness must vary according to the quantity of fuel consumed. Still the gas does not separate entirely: a portion remains mixed with the air in the upper part of the room, which contains about 1-78th; while that on the floor contains 1-19th. It is possible that this phenomenon may be due to gaseous mixtures, taking place in definite proportions, as well as to the effect of the difference of temperature in the different strata of air. The fact is somewhat analogous to the separation of metals, according to their density, in melting certain kinds of allays. The following case is quoted as confimatory of this view:

A gentleman tried to destroy himself by burning charcoal in his chamber. For this purpose he lighted a brazier and threw himself on the bed. Not finding that he became affected by the vapour, he for the time abandoned his design. Upon mentioning the circumstance to some friends, as if another party had been concerned, he learned from a professional man present that carbonic acid only produced serious effects to those placed on the floor of a chamber. A few days afterwards, this gentleman was found dead in his room, sitting at the foot of his bed, and a candle still burning on a table. It was evident that he had destroyed himself by charcoal vapour.

4. *On the nature of asphyxiating media.* The only data which exist on this subject are derived from the experiments of Orfila. The analysis of the vapour produced from charcoal, according to this chemist, varied with the degree of combustion; when this was *slow*, the carbonic acid formed about one-seventh of the mixture; when *rapid*, one-ninth: these results appear so paradoxical, that M. Devergie places but little reliance on them.

Charcoal vapour, when existing in large quantity, has a bluish tint, owing to the smoke mixed with it. This colour the author has found to disappear in twelve hours after the completion of combustion, and he thinks it may become still more rapidly dissipated. This fact, he observes, may be useful as a datum to determine the period at which asphyxia probably commenced in an unknown case. The vapour has likewise a peculiarly disagreeable odour, producing nausea and cephalalgia; it is chiefly perceptible when the charcoal is first ignited, and disappears so soon as the combustion has become very active. It probably has some influence in the production of asphyxia, and to it may be attributed the narcotic effects of charcoal. This charcoal vapour is heavier than atmospheric air; it reddens litmus, gives a white precipitate with lime-water, and extinguishes burning substances. In relation to the last character, it is necessary to observe, that in a state of admixture with air, it may produce asphyxia without extinguishing the flame of a lighted candle. Among the experiments performed, a cat and two birds were asphyxiated in an apartment, in which five candles were burning. This vapour never contains sufficient carbonic oxide or carburetted hydrogen to become inflamed by the approach of a candle.

5. *On the quantity of charcoal which is required to be burnt, in order to render a certain quantity of air deleterious.* Varin showed that animals perished in about three minutes, when plunged into an atmosphere containing about one-fifth of its volume of carbonic acid. But it is not necessary for asphyxia to ensue, that the atmosphere in a room, in which charcoal is burning, should acquire such an impregnation as this. There is obviously a great difference between a mixture of free carbonic acid and air, and a mixture containing carbonic acid actually produced at the expense of the oxygen of the air. Thus, in the two cases, the air may not contain more than ten per cent. of carbonic acid; but where this has resulted from combustion, the atmosphere will contain seven per cent. less of oxygen, and seven per cent. more of nitrogen, than in the case where free carbonic acid has been mechanically added to air. On the other hand, Varin proved that the strongest proportion in which carbonic acid could be mixed with air without rendering it injurious to respiration, is about two per cent. If only one-fourth of the oxygen be removed by combustion, it will contaminate the air with one-twentieth of carbonic acid, and render it asphyxiating.

Experiments were then performed to determine how much charcoal was required to be burnt, in order to remove one-fourth of the oxygen from a given quantity of air. In these experiments it was necessary to allow for the bulk of the charcoal; the wood from which it was made; the degree to which the charring process had gone on; the quantity of hygrometric water present, and the different proportion of salts entering into its composition. We need not detail these experiments, since, as the author allows, from the circumstances above mentioned, they can only be regarded as remote approximations to the truth. An answer to a question of this kind must therefore be a matter of pure speculation.

6. *How to determine the capacity of a chamber in which asphyxia has taken place.* This is rather a question for a surveyor. We are directed to multiply the length in feet by the width, and the product by the height, to obtain the measurement in cubic feet. The author lays great stress upon our making an allowance for any want of angularity in cupboards, closets, &c.

7. *How to determine the quantity of charcoal which has been consumed.* Charcoal in burning, leaves on the average about four per cent. or 1-25th of its weight of ashes. All we have to do is to collect the ashes and to multiply them by 25. Here we are met by the objection, that different kinds of charcoal yield different quantities; and we can test the accuracy of our inference only in those cases where some of the charcoal used happens to remain unconsumed. The author advises us to satisfy ourselves that there were no ashes already in the brazier, prior to the asphyxiating attempt being made.

8. *On the closure of apartments and its influence in the production of asphyxia.* It is a matter of general belief, that for asphyxia to take place it is necessary that the apartment in which the charcoal is burning should be perfectly closed. That this view is erroneous many cases on record clearly show. The perfect closure of a room is a circumstance highly favourable to the production of asphyxia; but it is not an indispensable condition. In the author's experiments the windows were loosely secured, and the chimney not perfectly closed. Persons have been repeatedly found asphyxiated in apartments, where there was a pretty free communication with the external air, by means either of a half-opened window or flue. We are then justified in affirming that this imperfect closure of outlets is unfavourable to the production of asphyxia, but that it does not render its occurrence impossible.

9. *On the influence of the situation of the person in the occurrence of asphyxia.* A case has already been related (3,) in which it was shown that the asphyxiating atmosphere had not the same intensity throughout. An individual, who escaped this form of death in a first attempt while lying on a bed, fell a victim to it when, on a second occasion, he placed himself on the floor. It has been proved, that during the actual combustion of charcoal, the air is equally impregnated with carbonic acid throughout; but that in the act of cooling this gas fell by its greater specific gravity to the lower part of the chamber. The following points may therefore be considered as settled: 1. That, whatever be the situation of a person, he will perish from asphyxia if the quantity of charcoal consumed was sufficient, *during combustion*, to render the atmosphere deleterious: 2, If two persons are situated, the one on the floor and the other three or four feet above that level, the latter may not be asphyxiated while the former may perish, provided the quantity of charcoal consumed was insufficient to render the air deleterious during its combustion, but still sufficient to impregnate with carbonic acid during the act of cooling the lowest strata of air.

10. *On the influence of sex, age, and profession, (!) on asphyxia.* All persons are not equally susceptible of the influence of charcoal vapour. Those who consume much of this kind of fuel, for domestic or other purposes, resist its effects better than others. According to the Police Registers of 1834-5, there were in Paris 360 cases of asphyxia by carbonic acid. Out of this number there were 19 cases of two persons together (men and women,) and only one case of two men together. There were only three instances out of these 19 cases, in which one of the two persons could be resuscitated, and in each of these the subject was a female. The number of females saved is greater than of males. In 184 cases of asphyxia, occurring during the year 1835, there were eighteen females saved out of seventy-three cases, or about one-fourth, and nineteen males out of eighty-three cases, or the proportion of about one fifth.

MM. Mayre and Ollivier have quoted cases which lead to a contrary inference, showing that there is a greater power of resistance to the effects of this gas in males than in females. M. Devergie pronounces these to be isolated facts, and prefers his own results.—*Annales d' Hygiene.*

[REMARKS.—Such is an abstract of M. Devergie's late researches on asphyxia from carbonic acid. Since we last adverted to this subject one or two cases have occurred in this metropolis which have given rise to considerable discussion, and it cannot be concealed that there is even now so great a difference of opinion in the profession, that probably no two medical witnesses would agree in their answers to some of the questions proposed by M. Devergie. There are many statements made by the author of this paper, to the correctness of which we cannot assent; and as we should regret to be the means of disseminating views which might give rise to much mischief, if implicitly relied on, we shall here offer a few remarks on some of the most disputable doctrines.

In the second query we find it stated, that the air by expansion, if unable to find a ready outlet, will exert a compressing force on the body, impeding respiration, and accelerating asphyxia. We can understand that the phenomena of this kind might ensue, if the man as well as the fuel were confined in a hermetically sealed vessel; but it seems to us impossible to admit, that in ordinary apartments, any such serious effects as the author imagines can take place.

In the third query, M. Devergie assumes from his experiments, that gases of different specific gravities mixed by heat, are capable of separating from each other to a greater or less extent on cooling, so that the heavier gas falls in great proportion on the soil or floor. To us this appears a material question in relation to death by carbonic acid; and the grounds on which such a statement is based require to be closely scrutinized.

The law of the diffusion of gases, now so well known to all chemists, has contributed to remove the old opinion, that carbonic acid from its weight had a tendency to collect and remain on the lowest levels. Gases are now known to mix readily on contact, whatever be their relative densities. Thus carbonic acid will rise into hydrogen, and hydrogen will fall into carbonic acid, although the difference of sp. gr. is as 22 : 1. There is no proof of this mixture of gases taking taking place in *definite* proportions: on the contrary, they are generally considered to mix in *all* proportions. Again, another fact commonly received by chemists is, that when once mixed, gases do not again separate from each other according to their specific gravities. M. Devergie, while he admits the principle of diffusion, affirms, that where this has been aided by heat, a partial separation takes place on cooling, so that carbonic acid and air, although mixed when heated, separated when cold. The single experiment which he adduces in support of his peculiar view is unsatisfactory. It would be impossible by such a method as that pursued by him to determine, with the least pretension to accuracy or certainty, the relative proportions of carbonic acid when mixed with air. This will be easily proved by trying the experiment with a mixture containing a measured quantity of pure carbonic acid. The case which he adduces is altogether inappropriate as an illustration. How are we to know that, in the first attempt at suicide, when the man was lying on the bed, the air of his apartment became sufficiently impregnated with carbonic acid to destroy him? yet it is here assumed, that in the two attempts there was an equally fatal contamination, and that the deceased only died in the second attempt, because he had placed himself on the floor!

We might stop here; but we are desirous of showing, not merely that M. Devergie's opinion is unsupported by his own experiments and reasoning, but that it is at variance with the most simple facts of almost daily occurrence. If on cooling, carbonic acid separated from air, as alleged by him, it is not to be expected that when the gases are once perfectly cold they would readily mix, nor that the heavy gas would again rise into the air and diffuse itself with a rapidity proportioned to the surface of contact, and yet that these phenomena do actually occur the most simple experiments will show. If a large glass vessel, having a diameter at the mouth of about five inches, with a depth of sixteen inches, be filled with carbonic acid and exposed to the access of air in a quiet and cool apartment, the whole of the gas will disappear from it in the course of a few hours. How is this to be accounted for if M. Devergie's position be true? If his opinion be correct, the carbonic acid should not leave the vessel. Let the capacity of its mouth be five times as great as that above mentioned, the gas will quit the vessel in a *few minutes*, from the greater extent of surface exposed. Now this is precisely the condition of M. Devergie's asphyxiating apartment: there is considerable extent of surface, and yet it is to be supposed according to him that the carbonic acid collects in larger proportion at the bottom of the room than it does at the top. If M. Devergie's statement were well founded, it is obvious, that in a calm state of the atmosphere the air in the streets of a crowded metropolis would be perfectly irrespirable.

In support of the erroneous view which we have just condemned, we sometimes find it stated, that carbonic acid exists in wells, on the floors of cellars and grottoes; and the Grotto del Cane near Naples, is often quoted as an instance where the gas is collected on the soil. But in all these cases it is forgotten that the source is permanent, and that the gas is continually issuing from fissures in the soil, or from decomposed matter.

To our minds, it is not proved satisfactorily that any separation of gases ever takes place, such as that above assumed; and while there is this want of evidence on the one side, there are numerous facts on the other, which render it certain that such a separation does not ensue, at least to any perceptible extent.

In the ninth query we have a practical (and in our view a most erroneous) deduction from the circumstances which we have just been considering. Thus it is assumed as probable, that of two persons in a small chamber where charcoal is burning, the one on the floor may perish, while another three or four feet above that level may escape, owing to this supposed separation of gases. We do not believe that such a case is likely to occur; and if it did take place, the result would probably depend upon whether the individuals were equally near the source of combustion. M. Devergie admits, and indeed proves, that the poisonous gas becomes equally diffused all over the room: that it may be insufficient to destroy life while thus diffused, but that it becomes destructive by slowly falling to the floor, and there forming a concentrated stratum. We have already said enough to show that if this be the case with charcoal vapour, it differs materially in its chemical and physical properties from those known to be possessed by carbonic acid. In our view, most people are killed by carbonic acid during the actual diffusion of the gas, so that the subsequent separation on cooling, even if it took place, would have very little influence in producing a fatal result. Besides, we must protest against the correctness of the inference so generally drawn at coroner's inquests in these cases, that because a person is found lying asphyxiated on a floor, he must therefore have been affected by the gas while in the recumbent posture. It is possible that he may have been sitting or standing, and have fallen asphyxiated. To analyze the stratum in which his head is found lying, and infer from the quantity of carbonic acid there discovered, that he has been attacked and has died in the recumbent posture, is absurd; since, unless the body be accidentally supported, every asphyxiated subject must be thus found.—*Eds. Brit. and For. Med. Rev.*]

---

*Remarks on the Tinctura Opii Ammoniata of the Edinburgh Pharmacopœia.* By J. H. Gilbert, Nottingham. Being a student in the laboratory of the Glasgow Royal Infirmary during the summer of 1839, it was occasionally my duty to dispense the *Tinctura Opii Ammoniata*,—a preparation, the apparently unchemical composition of which led me to suspect, that as far as the opium employed is concerned, it could be of no utility; it being well known that ammonia acts as a precipitant to morphia. It is true that a great excess of that alkali will, in some solutions of the salts of morphia, either prevent its precipitation, or redissolve it when precipitated. In order, therefore, to ascertain the fact in the present instance, I submitted several separate portions of the tincture to examination at Dr. Thompson's laboratory, where also I was a student.

Two ounces were first boiled in a flask with magnesia; the magnesian precipitate collected and boiled with alcohol; the alcoholic solution filtered while hot, and set aside in a warm situation to evaporate slowly: no crystals were deposited, and the residual matter, when tested, was found to contain no morphia.

Four ounces were next treated in a manner varying from the above. The liquid, previous to further treatment, was saturated with dilute acid; for when, as before stated, morphia has been held in solution by an excess of ammonia, I have found that, after expelling the greater part of that excess by gentle heat, and then saturating with dilute acid, a salt may be obtained; from the solution of which the morphia may be separated by the usual means. In the present case, however, no morphia was detected.

Having thus failed to detect that principle in the filtered tincture, the dregs of a known quantity were macerated for some time in water acidulated with muriatic acid;—the filtered liquid, after being digested with animal charcoal, was sufficiently concentrated on a water bath, and set aside, when a large quantity of crystals of muriate of morphia was deposited. A part of these were treated with ammonia, and the morphia tested in a separate state with a neutral solution of the perchloride of iron; and part, while in the state of muriate, was tested with iodic acid in the usual manner. As the dregs employed were not exhausted, nor the mother liquor treated with ammonia, an accurate quantitative result could not be obtained; but the amount of muriate of morphia was so great that it could not fall much short of the average produce of such a quantity of opium.

These experiments I had considered as conclusive. But, finding the new Edinburgh Pharmacopœia retained the old formula, and that the remarks made on the tincture by various writers on materia medica and pharmacy seemed to indicate previous experiment, I feared that either too small a quantity had been operated upon, or that some source of error had been overlooked. To obviate any such mistake, several portions, each of twelve ounces, were successively examined; in none, however, was morphia detected. As a check to the processes, several portions also of tincture of opium, (each of which would contain, by estimate, a quantity of opium equivalent to that in twelve ounces of the ammoniated tincture,) were treated in precisely the same modes as the ammoniated tincture had been. In these cases morphia was separated without difficulty; thus proving the efficacy of the processes.

It may be observed, that, before any of the tinctures were boiled with magnesia, the greater part of the ammonia and alcohol in the one, and of alcohol in the other, was expelled by gentle heat on the water-bath; as, in some cases, the alcohol in the liquid would be sufficient to take up the morphia after the decomposition of its salts by magnesia. The loss of ammonia caused no deposition of morphia.

These facts seem to show that, if, as I believe is universally admitted, the medicinal effects of opium are attributable chiefly, if not entirely, to the morphia which it coutains, the opium employed in this tincture might, with advantage, be omitted.—*Edinburgh Med. and Surg. Journ.*

---

*Physiology of the Iris.*—Mr. W. P. Brodribb relates, in the *London Medical Gazette* for May last, an interesting case, illustrative of the physiology of the iris. The patient, a young man 30 years of age, is completely *amaurotic* in his *right eye*, so as not to be able to distinguish light or darkness; the pupil of the eye is, however, but slightly dilated, and the iris is not much impaired.

On the other hand, vision in the *left eye is injured*, whilst the pupil is widely dilated and the iris perfectly immovable. When light is admitted into this eye, its iris remaining fixed, the iris of the amaurotic one contracts.

The case is one of disease of the brain, and the patient labours under complete ptosis of the left eye, with paralysis of all the parts supplied by the third pair of nerves of this side.

---

*Healthy Appearance of the Internal Surface of the Stomach.*—Very various are the accounts given by different authors who have written upon this subject, of the natural and healthy colour of the mucous membrane. It has been described as being white, grayish-white, grayish, reddish, grayish approaching to yellow and red, straw-coloured, &c. Billard, in whose opinion Dr. Hodgkin is inclined to place some confidence, states it to be a dead milky-white. According to Buison and Bichat, the colour is of a deep red, and Sabatier and Habicot describe it as of a reddish-purple and deep purple. Gavard Boyer, Soemmering, Chaussier, and Adelon make it of variable shades between red and gray. Rousseau who derived his opinion from the examination of the bodies of criminals dying by the hands of the executioner, (by the guillotine, we presume,) states that the colour of the gastro-intestinal canal is white, or white faintly tinged with red. Dr. Yelloly states, that in various opportunities which he had of examining the human stomach soon after death, in such parts of it as were free from vascularity, it had usually a light coloured tinge, but gives it as his opinion, that from the analogy of the mucous covering of the mouth and fauces, and of the urethra, it is probable that when circulation is going on in the stomach, its inner surface is of a pale red hue, arising from vessels so minute as to give an uniform colour, without any appearance of distinct vascularity. We are ourselves rather disposed to agree with M. Hippolyte Cloquet, who describes the usual appearance of the membrane as being of a reddish white and mottled, (comme marbreé,) but we must observe that this diversity of opinion as to a fact so evident to the senses, could only have arisen from the varying appearances of the membrane presented to the several observers under different circumstances of disease, or from the effects of certain physical agents acting during the last moments of life. The manner of death would appear to exert considerable influence; the presence of aliment recently taken into the stomach causes a decided red tinge throughout the membrane; extremes of cold and heat, according to Beaupré, are also productive of a like effect in the mucous membranes generally, and the stomach has been observed to take a decided tinge from the various medicines administered shortly after death.—*American Jour. of the Med. Sci.*, from *Brit. and For. Med. Rev.*

---

*On Croup and its Cure.* By Dr. Grahl, of Hamburgh.—Dr. Grahl calls attention to the employment of arm-baths in croup, the use of which has already been advocated by Dr. Dorfmüller in the fifty-first volume of Rust's Magazine. They are indicated in the commencement of the stage of exudation, the existence of which is rendered evident by the difficult respiration. He regards the employment of leeches in this stage as of no avail, and the administration of sulphate of copper and emetics as likely to increase the exudation. His recommendation is that the arms of the patient be placed in a vessel sufficiently deep to admit to a hand's breadth above the elbow-joint, and filled with water as hot as it can be borne. A cloth should now be thrown over the head of the patient, which, falling down around the edges of the bath retains the vapour, and this the patient should be allowed to respire for a quarter of an hour together, repeating it at short intervals. The first application usually induces some degree of moisture in the Schneiderian membrane, and diminishes the dyspnœa. As it is repeated the cough usually loses its hoarse tone, and the patient expectorates the exuded lymph. In cases where the symptoms are extremely urgent, calomel in large doses should be given, and a blister applied to the throat, but in the great majority of cases the employment of the arm-bath is all that is necessary.—*Brit. and For. Med. Rev., from Zeitschrift für die gesammte Medicin.*

---

*Communication of an Intermittant Fever from a Mother to her Child.* By Dr. Brunzlow, of Brandenburg.—The mother suffered throughout nearly the whole of the last seven months of her pregnancy with her first child from tertain and then from quartan ague. The child was born very weakly, and after some weeks of more or less illness the mother noticed that it appeared much worse on some days than on others. On closely watching it, the author found that every fourth evening the infant became very cold and had its nails quite blue, and that then a considerable degree of heat came over the whole body, and was followed by slight sweating. On the next morning all these symptoms had disappeared, and for the next two days the child remained perfectly well. The evidence of its being actually affected with quartan ague was further strengthened by its being slowly cured by the administration of quinine.—*Ibid, from Medicinische Zeitung.*

---

*Poisoning of Leeches by the Blood of the Sick.* By Stefano Grandoni.—In a paper containing experiments to prove that portions removed from leeches are not reproduced, the author relates the following interesting case:

In the beginning of May there was brought to the Brescia Hospital for Males, a butcher, who had contracted malignant carbuncle in cutting up an ox that had died of that disease. Among the means employed were 120 leeches, which were to be applied in three divisions to his right arm, all of which died on being taken off the skin of the patient. Our chemist being informed of the case, though he did not doubt that the death of the leeches proceeded from the blood which they had sucked and were full of, had several other lively and strong leeches applied, and these also immediately fell off dead. This having occurred in the calamitous time of the cholera, curiosity was excited to know whether leeches would also die from the effects of the blood sucked from cholera patients, and it was found on good information that this had actually occurred in the cholera hospital.—*Ibid, from Annali Universali di Medicina.*

---

*On the Treatment of Acute Articular Rheumatism by large doses of the Nitrate of Potash.* By M. Aran.—This is a very long paper, detailing the results of twelve cases of acute rheumatism treated by MM. Gendrin and Martin Solon. Of these, ten were males, two females, from the ages of sixteen to forty; the stage of the disease varying from the second to the fifth day, and the previous treatment having been unimportant. The nitrate of potash was administered in some demulcent ptisan, the mean quantity taken by each patient being *an ounce and two scruples per diem*, and twelve ounces throughout the whole duration of the disease. The mean duration of the disease was eight days from the commencement of the treatment, and rather more than twelve from the period of invasion. The usual effects were very abundant perspirations; occasionally copious stools, but without colic; and free secretion of urine. The digestive organs were unimpaired in their functions; only one patient vomited; the pulse was depressed; and the pain in the joints rapidly diminished. In three cases complicated by endocarditis or pericarditis, these affections rapidly subsided, though no other remedies were employed. Only two patients suffered a relapse, and they were again cured by the same means.

[This treatment is not new, for Brocklesby and White, in this country, towards the close of the last century, gave nitre from ten drachms to two ounces in the twenty-four hours, and spoke highly of its efficacy both in acute and chronic rheumatism. However the mean duration of the disease under its use does not appear to be less than is obtained by other means. Dr. Hope, who trusted to calomel and opium, states the usual duration to be eight or ten days, and we have frequently seen severe cases rapidly cured by colchicum and opium. Bouillaud, who employs free bleeding, gives one or two weeks. Dance, who gave the tartarized antimony freely, forty to sixty days. The nitre is evidently preferable to the two latter.]—*Ibid, from Journal des Connaissances Médico-Chirurgicales.*

---

*On the Pathology of Plica Polonica.* By Dr. Bidder, of Dorpat.—In a paper on the structure and physiology of the human hair, the author has communicated the following interesting observations on this disease, which he had an opportunity of making during a residence of several weeks in a district in which it was very prevalent, though not in its severest form.

In none of the cases examined did the matting of the hair extend to the scalp, but for a distance of between half an inch and an inch from it all the hairs, however matted in the rest of their length, were perfectly normal. It might be imagined indeed, that healthy hair had in these cases begun to grow again, and that in an earlier stage of the disease, the whole had been matted together; but the same appearance having been observed in at least twenty individuals placed under a variety different circumstances, proves that it is a constant and essential fact. Moreover, since the scalp beneath the matted hair was perfectly healthy, exhibiting neither redness nor swelling, nor increased sensibility, it is impossible to refer the morbid appearances in the hair to disease of its matrix. It must rather be held, that in consequence of a morbid process commencing in a defined part of their constituent fibres, the hairs stick together in bundles of various sizes, and become individually so loosened in their texture, that they split up into smaller and finer portions. The frequent occurrence of such fine hairs, as well as that of much coarser hair in the plica, can scarcely be otherwise explained.

A fact still more remarkable, and affording a still stronger proof of the vitality of the hair, is a process of separating or shedding which occurs in some cases of plica. The author saw two persons from whom plicæ had lately fallen off spontaneously, without leaving any part of the head bald; and indeed this occurrence is regarded by the people of the district as a favourable, though a rare symptom in the disease. In two others he was able to trace the mode in which the separation takes place.

In these, there formed at the limit between the diseased and healthy hair, in the tuft that was matted beyond a certain distance from the scalp, a deep groove which completely encircled the tuft as if a string had been tied round it. It formed a very distinct boundary between the two parts of the tuft, and as it went on, the constriction becoming gradually deeper, a part of the hairs separated, and the whole plica was held by only the remainder of those by which it was originally constituted, and at last it entirely fell off. The whole process bore a remarkable resemblance to that of the casting off of other diseased parts, such as occurs, for example, in gangrene; and the whole series of the phenomena are explicable only on the belief that they depend on an action occurring in the hair itself.—*Ibid, from Müller's Archiv.*

---

*On Horny Excrescences on the Eyelids.* By Dr. F. A. V. Ammon.—Professor Stromeyer, of Erlangen, had the kindness to send me a drawing of a horny growth, with the following description: "This horn has existed ten weeks, and still goes on growing; six weeks since the excrescences on the nose formed, and these are now ready to fall off. The horny mass does not proceed from a follicle, but was probably secreted by the vascular skin at its base. It gives no annoyance; the closing of both the eyelids is effected only imperfectly from want of tone, and the conjunctiva is somewhat loose. The patient is a woman, seventy-one years old." Thus far Stromeyer.

To this interesting communication I may add the following. I have twice observed horny growths on the eyelids. The cases were as follows: A stocking-maker, forty years old, healthy and strong, had a horny growth, three lines large, on the right upper eyelid, a few lines from its ciliary margin. It had grown gradually, and turned downwards. The patient believed that it came from a wart; and he came to me only because he had lately often struck the growth, and given himself pain. I cut it off with scissors, going rather deep into the healthy skin, and the wound bled considerably for some time. When it had ceased bleeding I touched it with nitrate of silver, and in twelve days it was completely healed. Two years have since elapsed, and the disease has not reappeared.

The most accurate examination detected nothing more than that the horn was a dark cartilage-like substance, uniform from from its base to its apex, and consisting of several close lamellæ. Where it was connected with the skin, there was seen an intermediate substance like a thickened cutaneous gland.

In the second case, I observed the disease in a woman fifty years old, who had ceased menstruating for some time, and had had warts on many parts of her body successively, which, however, were always removed by the remedies she appplied. From a wart on the left upper eyelid, a horny excrescence had gradually formed, which was upwards of four lines high, inclined to one side, and smaller at its apex than at its base. It gave no pain, but it often itched, and when the woman scratched it, it bled. I extirpated it with scissors, and it has not been reproduced.

As far as I know, among the cases of horny excrescences described by authors, there is none of a *cornu cataneum* of the eyelids.—*Ibid, from F. A. V. Ammon's Monatsschrift für Medicin.*

---

*Foisoning by Solution of Acetate of Lead—Lead found in the Urine.*—A young girl of good constitution, driven by despair to suicide, took about an ounce of acetate of lead in solution. She was almost immediately seized with collapse and syncope, and afterwards with vomiting and convulsions. Sugared water, sulphate of magnesia, and sulphate of soda were given, but she died in twenty-five hours. She voided a large quantity of urine, which M. Villeneuve sent to M. Orfila. Carbonized, treated by nitric acid, and submitted to the tests of the salts of lead, this urine afforded a sensible quantity of lead.—*Ibid, from Journal des Connaissances Médico-Chirurgicales.*

---

*Antimony in the Urine.*—At the sitting of the Royal Academy of Medicine, Dec. 8, 1840, M. Husson stated that he had given a scruple of tartar emetic to a potient affected with pneumonia in twenty-four hours. It produced neither stools nor vomiting, and on the urine being examined by M. Orfila, with Marsh's apparatus, it afforded the antimonial stains in great abundance.—*Ibid, from Archieves Gén. de Médicine.*

---

*On the Employment of Arsenious Acid in Phthisis Pulmonalis.* By M. Trousseau.—M. Trousseau is now making trial of the powers of arsenic in phthisis at the hospital Necker; a means, however, not new, as Dioscorides employed fumigations of the sulphuret of arsenic in the same disease. During some few months M. Trousseau has submitted eight patients to the action of this agent. In four affected with diarrhœa, the disease continued its progress; it was in an advanced stage, and death occurred in the usual manner. In four others, notwithstanding vast caverns, and symptoms which announced approaching death, the symptoms amended under the influence of arsenious fumigations, the general health improved, appetite returned, digestion was good, emaciation checked, and the cough and expectoration diminished.

A sheet of white paper is dipped in a solution composed of one part of arseniate of soda and thirty parts of water. The paper is then made into little cigars of the length of a finger, and the patient is directed to smoke one or even two daily, in such a manner that the fumes may pass into the lung. This is readily accomplished, by the patient inspiring the moment the fumes enter the mouth. This inspiration of arsenious vapour at first causes slight cough, but after some time both cough and expectoration are much diminished.

This means, though it has not as yet cured phthisis, deserves a trial. In one case of chronic catarrh with emphysema, it rapidly removed symptoms of suffocation. Careful experiments should be made with this remedy, for phthisis is a disease hitherto so incurable that anything which affords any probability of effecting permanent good is worthy of investigation.—*Ibid, from Bulletin Général de Therapeutique.*

*On certain Anthelmintics in use in Abyssinia.* By Dr. AUBERT.—Dr. Aubert, who has resided some time in Abyssinia, states that the whole medical practice of the natives consists in the employment of three indigenous vegetable anthelmintics called Cusso, Bisenna, and Abbatsjogo; and that tænia is so common among the inhabitants as to be considered almost natural. Bruce remarked that almost all Abyssinians were affected with ascarides; but as the Abyssinian word applies to either species, and Bruce was not a physician, it is probable he confounded the two, especially as the cusso, which he praised as a remedy against these worms, Dr. Aubert finds used in tænia.

The cusso is the Banksia Abyssinica of Bruce, the Hagenia anthelmintica of Willdenow, one of the species of the genus Saponaria of Linnæus. M. Kunth, an able botanist, has recognized the flowers as a new genus of the Rosaceæ. About two drachms of the flowers in powder are taken in an electuary of honey, and ordinarily in about twenty-four hours the patient passes the worms, but without purging. The whole of the worm is rarely expelled; but the patient follows his usual occupation, and after a time, as the medicine is not unpleasant and does not cause griping or purging, the dose is repeated. Dr. Aubert became a subject of tænia whilst in Abyssinia, and by taking aperient medicine after the cusso, he completely relieved himself of it.

The bisenna is a species of conifera much resembling the Juniperus Virginiana of Linnæus. The pulverized bark is employed, but not so generally as the cusso, as it causes irritation, colic, and in some cases enteritis.

The abbatsjogo resembles the Ixia bulbocodium of Linnæus. It is less employed than either of the former species.—*Ibid, from Bulletin de l'Académie Royal de Medécine.*

*On Tincture of Iodine as a topical Application to Phagedenic Chancres.* By M. RICORD.—After a trial of all the most common topical applications recommended in the treatment of phagedenic chancre, M. Ricord finds them all frequently inefficacious, and from none has he obtained such prompt and happy results as from the tincture of iodine. He has employed this tincture during the last three months, and almost constantly obtained a prompt modification of the ulcerated surfaces, which soon lose their phagedenic character. This has occurred in many cases. The action of the iodine was particularly evident in a patient who had an open bubo, the ulceration being phagedenic and very extensive. Various means were employed during two months without success, when the tincture of iodine alone so modified the ulcerated surfaces that their extent visibly diminished from day to day. The iodine of potassium had been given in this case without effect, but its use was continued conjointly with the tincture of iodine as a topical application. The cure was perfect in less than a month.—*Ibid, from Bulletin Général de Thérapeutique.*

*Great Hæmorrhage after Division of the Genio-glossi Muscles for Stammering.* By M. GUERSENT, Jr.—M. Guersent performed the above operation at the Children's Hospital on a child twelve years of age. The hæmorrhage at the time of the operation was very slight, but the next day it came on to a large amount, and was checked by charpie dipped in a solution of alum. On the third day the hæmorrhage recurred, and was checked by the same means. Fourth day, no hæmorrhage, cold lotions, ice in the mouth. Fifth day, hæmorrhage came on with the greatest violence, but was stopped by the actual cautery. Sixth day, no hæmorrhage. Seventh, it recurred, and the red-hot iron was applied seven times without arresting it; compression, by means of pledgets of charpie steeped in a solution of alum checked it after several hours. The compression was continued on the following day, and permanently suppressed the bleeding. The state of this child excited the greatest solicitude. The weakness and pallor indicated such pressing danger that M. Guersent had determined to tie the lingual arteries if the compression had not effected his object.—*Ibid, from Bulletin Général de Thérapeutique.*

*New System of Bandaging.* By M. RIGAL, of Gaillac.—The object of this memoir, which was read at the Royal Academy of Medicine in November, 1840, is to point out the advantage obtained by the use of strips of Indian rubber in the place of common ligatures and bandages, made elastic by means of the same material, in place of the common roller. The author has used them for the last two years, and speaks very highly of their power of maintaining parts in apposition during the most varied movements of those parts. He particularly instances hare-lip, and the operations for the restitution of lost parts. The elastic bandages are also said to be very useful in maintaining oblique fractures of the lower extremities in perfect apposition, opposing a force in constant operation to the irregular action of the displacing muscles. In this way also they may assist in the replacement of parts after tenotomy.—*Ibid, from Revue Médicale.*

*Ectropion cured by Autoplasty.* By M. Berard.—M. Bérard presented to the Academy a young butcher whose inferior eyelid, all except the free border, had been destroyed by a malignant pustule. After having arrested the progress of this terrible disease, M. Bérard sought to repair the loss of substance and prevent greater depression of the free border of the eyelid already affected by ectropion. A portion of integument was taken from the temporal region and applied just below the inferior eyelid. The superior border of this slip became united to the inferior border of the eyelid, and its inferior border to the superior edge of the malar integuments. The whole was maintained in apposition by simple agglutinatives. Four months after the operation, the wounds are cicatrized, the new eyelid is perfectly vital, and of a tint precisely similar to that ordinarily seen in the situation which it occupies —*Ibid, from Bulletin de l'Académie Royale de Médecine.*

*Iodine in Opacity of the Cornea.* By Dr. Lohsse.—The case in which this remedy was successfully employed was one of opacity of the cornea consequent on syphilitic ophthalmia, and so considerable as almost completely to destroy vision. The iodine was given internally, and from four to six drops of the following collyrium were let fall into each eye three times a day:

℞. Iodini, gr. j.
Potassii iodidi, gr. ij.
Aq. dest. ℥vj. M.

Afterwards this was exchanged for an ointment consisting of iodine gr. jss, Iodide of potassium, ℈j, and lard, ℥ss, of which a small portion was once or twice a day put between the eyelids. The cure was perfected in three months.—*Ibid, from Medicinische Zeitung.*

*On Congenital Dislocation of the Femur.* By M. Bouvier.—At the sitting of the Royal Academy of Medicine, Jan. 26, 1841, M. Bouvier presented two preparations illustrative of the two principal forms of congenital dislocation of the femur. In one the head of the femur is received in a superficial cavity above and behind the cotyloid cavity, which latter is reduced to very small dimensions. The capsule embraces both the new and old cavities, and is strongly applied around the femur, the movements of which it limits, and does not allow the head to be brought opposite the cotyloid cavity. The subject was a female seventy-six years of age, and the dislocation was said to have occurred when she was four or five months old, but the probability appeared that it was congenital.

The second specimen was taken from a woman fifty-three years of age, and in this no point of contact exists between the head of the femur and the ilium, which were attached to each other by the capsular ligament and muscles alone. This disposition of parts occurred on both sides, and we know that it is recognized in congenital dislocations alone, and especially in those which affect both sides. The head of the femur is lower and more behind than is usual in iliac luxations, and is only a few lines before the ischiatic notch, and very near the great sciatic nerve. Entirely covered by capsular ligament, the head of the femur rests on a cellulo-fibrous cushion, which here covers the surface of the ilium, and which is united to the capsular ligament by a sort of lax cellular membrane. The round ligament remains, much elongated, flattened, and partly confounded with the capsule. The head of the femur is almost of its normal size, while scarcely a vestige of the cotyloid cavity remains, and this of a triangular form, as is usual in most similar cases. Several of the muscles have undergone important changes, but none in such a manner as to limit the motions of the limb, all of which, with the exception of extension, were almost unaltered.—*Ibid, from Bul. de l'Acad. Roy. de Med.*

*Statistics of Amputation performed in the African Army, in Hospitals and the Field, during the years* 1837-8-9. By Dr. Guyon.—The number of amputations performed in the above years (the campaign of Constantine in 1837 excepted,) was 63, namely :

| | |
|---|---|
| Disarticulation of the shoulder-joint | 6 |
| " " elbow | 2 |
| " " wrist | 6 |
| " " knee | 1 |
| " partial of foot | 1 |
| " tarso-metatarsal | 1 |
| Amputation of the thigh | 16 |
| " " leg | 7 |
| " " arm | 15 |
| " " fore-arm | 8 |

Of these 63 patients, 46 were cured, 17 died. As, however, four died from circumstances scarcely connected with the amputation, the proportion of deaths may be stated as 1 to 11. This result is much more favourable than that during the siege of Constantine in 1837, for of 10 amputations performed at Médéah, only 1 survived, and of 62 at Blidah, 39 died.

Of the 63 operations referred to above, 44 were performed immediately, 19 secondarily. The former gave 32 cures, 12 deaths; the latter 14 cures, 5 deaths. Thus the proportion of cures after secondary amputation was not less satisfactory than that after immediate.—*Ibid., from Gaz. Med. de Paris.*

*On Compression in the Treatment of Mammary Abscess.* By MM. Trousseau and Contour.—This is a very long paper, illustrated by eight cases, to show the utility of compression of the breast by means of strips of diachylon plaster, an inch broad and several feet in length, carried completely round the body so as to produce regular and methodical compression of the whole breast. The conclusions of the authors are, 1, that this compression may be employed in all forms of inflammation of the breast in nurses; 2, it will sometimes cure when used at the commencement of the inflammation; 3, during suppuration it will not arrest the progress of the formation of pus, but it immediately relieves the pain; 4, compression should be made twenty-four or forty-eight hours after the abscess has been emptied; 5, under its influence the pain ceases, the walls of the abscess unite, fistulæ are cured, and a few days in general complete recovery. Continued for a time it prevents relapse.—*Ibid., from Journ. des Con. Med. Chirurg.*

*Hemorrhage after Delivery arrested by a new Method.* By Dr. Hecking, of Coblenz.—A weak and delicately made woman, soon after she had been quickly and easily delivered of twins, began to have more than usual hemorrhage from the uterus. Permanent pressure on the abdomen, with friction over the uterus, and repeated dashing with cold water were employed, but they were all ineffectual, and the hemorrhage grew more rapid. The author therefore introduced his hand into the uterus (after having dipped it into cold water,) and doubling his fist endeavoured, first by rubbing the parietes, and then by compressing them against his other hand, which was placed on the walls of the abdomen to bring on contraction of the uterus. But this plan also was altogether useless; the uterus, still remaining distended, was every instant filled with fluid and coagulated blood. After all attempts had proved vain, and when the patient's danger was now extreme, the author took a sponge dipped in cold water, and then squeezed it on the internal surface of the uterus, so as completely to wet it all over, at the same time maintaining the external pressure. As often as the sponge became covered with coagulated blood, it was taken away, washed with cold water, and again introduced as quickly as possible. By this means several times repeated (for in the powerless condition of the patient it was not a difficult thing to do,) the hemorrhage was at last arrested, and the woman's life saved.—*Ibid., from Casper's Wochenschrift.*

*Hernia of the Uterus, and Cæsarean Operation: Mother and Child saved.* By M. Ledesma, of Salamanca.—On the 26th of January, 1839, a female named Ramus, 24 years of age, of good constitution, having had six children, stated that some time before her marriage she had an inguinal enterocele on the right side. When the seventh pregnancy had advanced to the third month, she suddenly perceived an unpleasant dragging sensation at the bottom of the left side of the abdomen; the abdominal tumour disappeared, and she voided the some blood by the vagina. Something unusual, hard, and painful on pressure was now discovered in the site of the inguinal hernia. She frequently attempted reduction in vain. Seven weeks afterwards she perceived evident movements. The tumour descended from the bottom and right side of the abdomen on the thigh of that side, distending the right labium pudendi, falling on the pubis and crossing towards the left thigh; it was 22 inches in extent, 25 in circumference at its middle, and 22½ at its junction with the abdomen. It was fluctuating, and on displacing the liquid, a hard body was perceived which appeared moveable in the liquid. The neck of the uterus could not be found in the vagina. At seven months the sounds of the fœtal heart, and the placentar souffle when examined by the stethoscope were perfectly distinct from the superficial position of the uterus. M. Ledesma judged by the souffle that the placenta was inserted to the left and a little anteriorly: the sound was clear and very circumscribed. Attempts at reduction were useless. On the 6th of July she experienced her first pains. On the 7th the membrane broke, and the waters were discharged by the vagina. Reduction again attempted in vain. Hysterotomy was practised, with care to avoid the left lateral part of the tumour. A great quantity of blood followed the incision of the uterus. The head of the fœtus was against the cervix, the surgeon seized the feet which were at the fundus and forcibly withdrew the child. The placenta was at the place foreseen. After the uterus was emptied a tepid bath was prescribed, which excited uterine contractions and arrested all hemorrhage. Reduction of the uterus was thought to be impossible, and it was left externally.

July 12. The lochia take the rout of the vagina. After some accidents, slight in comparison with the severity of the operation, the patient's health is re-established. The wound suppurated more than a month.

July 13. The catamenia reappeared.

August 11. Convalescence is complete. The tumour has considerably diminished. The infant was well, but died some time afterwards. *Ibid., from Gaz. Med.*

*On Excision of the Elbow-joint.* By M. Roux.—Since 1812, M. Roux has performed excision of the elbow-joint eleven times, the whole of the articulation having been removed in every case. Of these eleven patients three have died, six reaped every benefit which could arise from the operation. The patient operated on in July, 1839, can flex and extend his arm imperfectly, while a few fistulæ still remain in the patient operated on last November. M. Roux formerly practised the operation of Moreau, making an external and internal vertical incision, and one transverse below the olecranon, but now he only makes an external and a transverse incision, the triangular flaps thus formed being quite sufficient when turned back to expose the articulation for removal, while the subsequent union is much more rapid than when an internal incision is also made.—*Ibid.*, *from Bull. de l'Acad. Roy. de Med.*

*On the improvement of the defects in the refractive power of the eye, by Myotomy.* By Dr. Kuh, of Breslau.—M. Jules Guerin, Mr. Phillips, and the author, have all divided the muscles of the eyeball in the hope of remedying short-sightedness by diminishing the amount of the muscular pressure on the exterior of the eyeball, by which its axis is lengthened. Philipps has divided the inferior oblique, an operation which the author thinks must be perfectly useless. Both he and M. J. Guerin have divided the straight muscles, either the superior or inferior recti; or, in different operations, all the recti of the same eyes. The author's first patient, after the division of all the recti muscles, could see to read as well at a distance of nine inches as before the operation he could at a distance of four inches. In the second case, in which the external and internal recti alone were divided the result was null; he could see no better than before the operation.—*Ibid.*, *from Casper's Wochenschrift.*

*Cure of an ununited fracture of the Humerus by a Seton.* By M. Jobert, of Lamballe.—A robust man, about forty-five years old, received in a fall a fracture of the humerus, complicated by a small wound at the seat of the fracture. The arm was placed in an unmoveable apparatus during a month. At the end of this time the small wound still suppurated, and there was no trace of consolidation. During two months longer the ordinary apparatus was applied, but did not hasten consolidation. M. Jobert then passed a seton between the two fragments, but instead of leaving it five or six weeks, as Physick and others have done, he only let it remain eight days. A month afterwards consolidation was completed. Experience has shown that in some cases, the seton has left after its passage organized fistulæ incapable of cicatrization, which maintain the mobility of the bone. Sometimes the passages border on portions of bone denuded by the seton. But when the seton is only left during eight days, it irritates the periosteum, and the inflammation of this latter brings the deposit of a sufficient quantity of osseous matter to effect consolidation.—*Ibid.*, *from Gaz. Med.*

*New mode of treating Orchitis.* By M. Dieulafoy, Surgeon to the Hotel-Dieu at Toulouse.—The following treatment has been em-employed by this surgeon in more than thirty cases, always with success and without accident: "with the left hand," says he, "I embrace the testicle at its superior part, and thus strongly stretch the scrotum at the inferior part of the organ, and thrust in a very sharp bistoury perpendicularly. Before the instrument is withdrawn it is necessary to enlarge the opening to allow the free exit of fluid. It is necessary to penetrate the tunica vaginalis, or the puncture is without effect. . . . Treated thus the duration of orchitis is from eight to ten days. . . . . I think this is the sole means the surgeon need employ in treating gonorrhœal orchitis. We need not fear to pierce very deeply, and wound the body of the testicle. Galbois has proved that wounds of the body of this organ are not dangerous, and are not followed by any accident."—*Ibid.*, *from Jour. de Med. et de Chir. de Tou.*

